# Serum sclerostin and irisin as predictive markers for atherosclerosis in Egyptian type II diabetic female patients: A case control study

**DOI:** 10.1371/journal.pone.0206761

**Published:** 2018-11-07

**Authors:** Mona Kamal Saadeldin, Shereen Saeid Elshaer, Ibrahim Ali Emara, Mohamad Maged, Amal Kamal Abdel-Aziz

**Affiliations:** 1 Department of Biochemistry, Faculty of Pharmacy (Girls), Al-Azhar University, Cairo, Egypt; 2 Department of Biochemistry, National Institute of Diabetes and Endocrinology (NIDE), Cairo, Egypt; 3 Faculty of Biotechnology, October University for Modern Sciences and Arts, 6^th^ October City, Cairo, Egypt; 4 Department of Pharmacology and Toxicology, Faculty of Pharmacy, Ain Shams University, Cairo, Egypt; East Tennessee State University, UNITED STATES

## Abstract

Diabetes mellitus represents a major independent risk factor for developing fatal cardiovascular diseases (CVDs) presumably through accelerating atherosclerosis; the underlying cause of most CVDs. Notably, this relative risk is reported to be higher in women than men. Endeavors directed towards identifying novel reliable predictive biomarkers are immensely thereby urged to improve the long-term outcome in these diabetic female patients. Sclerostin (SOST) is a Wnt signaling antagonist whereas irisin is a muscle-derived factor released after exercising which enhances browning of white adipose tissue. Emerging lines of evidence hint at potential crosstalk between them and CVDs. The present study aimed to assess the serum levels of SOST and irisin in Egyptian type 2 diabetic (T2DM) female patients with and without atherosclerosis and explore the possible relationship between both markers and other studied parameters among the studied cohorts. In this case-control study, 69 female subjects were enrolled; 39 type 2 diabetes patients with atherosclerosis (T2DM+ATHR), 22 type 2 diabetes patients without atherosclerosis (T2DM-ATHR) and 8 healthy controls. Their serum levels of SOST and irisin were assessed using ELISA. Significant increase in SOST levels were found in T2DM+ATHR compared to T2DM-ATHR and control (259.9 ±17.98 vs. 165.8±13.12 and 142.0±13.31 pg/mL respectively, P<0.001). Conversely, irisin levels were significantly lower in T2DM+ATHR (P<0.001) and T2DM-ATHR (P<0.01) compared to the control group (32.91±2.545 and 58.55±13.19 vs. 473.6±112.7 pg/mL). Interestingly, significant correlations between the levels of SOST and both irisin and fasting blood glucose were noticed in T2DM+ATHR group (r = 0.3754 and 0.3381 respectively, P<0.05). In conclusion, to the best of our knowledge, this study is the first to demonstrate the correlation between SOST and irisin levels in atherosclerotic T2DM female patients implying their potential implication in diabetic cardiovascular pathophysiology and supporting their use as reliable diagnostic/prognostic biomarkers for monitoring and preventing CVDs progression of T2DM female patients.

## Introduction

Over the last three decades, the number of diabetic patients has quadrupled worldwide. It has been reported that almost 1 in each 11 adults are diabetic patients, 90% of whom suffer from type 2 diabetes mellitus (T2DM)[1]. Diabetes mellitus (DM) inflicts a lot of complications that are categorized into microvascular and macrovascular complications [2], among which is atherosclerosis which is one of the main pathological manifestations in macrovascular diseases [3]. While in the general population, women were deemed to possess the “hormonal protective advantage” from cardiovascular diseases (CVDs), such relative protective effect is less pronounced upon developing DM. Indeed, after adjusting for other risk factors, DM was demonstrated to increase the risk of CVDs in both women and men by 3 to 4 times and 2 to 3 times respectively [4]. Hence, identification of novel reliable predictor biomarkers is indispensible for monitoring and precluding the progression of atherosclerosis in T2DM female patients.

Atherosclerosis induces improper proliferation of vascular smooth muscle cells (VSMCs) leading to atheroma plaque formation and stiffening of the arterial and vascular tissues [5]. The VSMCs migration, survival and proliferation is greatly controlled by the canonical Wnt signaling pathway [6–8]. New evidences show that cardiovascular pathophysiology is linked to the Wnt signaling pathway [9] whose activation encourages differentiation of mesenchymal stem cells (MSCs) to osteoblasts and myoblasts, however, inhibits differentiation into preadipocytes. Canonical Wnt signaling establishes a balance among myogenesis, osteogenesis and adipogenesis [10]. Sclerostin (SOST) is an endogenous antagonist produced predominantly by osteocytes, and studied as an important regulator of canonical Wnt pathway in the metabolism of bone [11,12]. At the molecular level, Wnt signaling represses adipogenesis via inhibiting CCAAT/enhancer-binding protein-*α* (CEBP*α*) and peroxisome proliferator-activated receptor-*γ* (PPAR*γ*) and halts the thermogenic program via suppressing PPAR*γ* coactivator-1*α* (PGC-1*α*) [13]. Subsequently, SOST induces adipogenesis [14] Scientists highlighted irisin because its presence confirmed a benefit of the physical exercise on fat, especially because it is looked at as a likely new treatment for T2DM and obesity [15] and because it is one of the inflammation-related factors whose expression is significantly affected in T2DM which is not simply a disease of hyperglycemia, but also is an inflammatory disorder [16]. Irisin is a recently recognized myokine produced directly into the blood circulation during exercise [17] and is secreted in response to PGC-1*α* activation; this hormone induces browning of white fat cells. This brown fat reduces body weight, increases total body energy expenditure and increases insulin sensitivity (7,8). Importantly, irisin evidently guarded against atherosclerosis in murine models via thwarting oxidized LDL-induced vascular inflammation and endothelial dysfunction. Accordingly, irisin is foreseen to be a promising candidate for treating several metabolic disturbances and CVDs [18,19].

Taken together, this study aimed to assess the levels of SOST and irisin in T2DM female Egyptian patients with and without atherosclerosis to correlate their levels with atherosclerotic events in T2DM patients and explore the possible relationship between both markers and among other studied parameters; fasting blood glucose, HbA1c, lipid profile, fasting insulin and Homeostasis Model Assessment (HOMA) in the selected Egyptian population in order to investigate the possibility of using it as a prognostic marker for the progression of the cardiovascular complications associated with the disease.

## Subjects and methods

### Subjects

#### Subject recruitment and classification

This is a case-control study that enrolled a total of 69 female subjects. According to the criteria of the National Diabetic Data Group (WHO criteria), they were divided into 61 type 2 diabetic patients and 8 healthy subjects; matching the same age and socioeconomic status. The diabetic patients were further divided according to the criteria defined by the American Heart Association, 2015 into 39 type 2 diabetics with atherosclerosis (referred to as T2DM+ATHR) and 22 type 2 diabetics without atherosclerosis (T2DM-ATHR). This study was approved by the ethical review board of the National Institute for Diabetes and Endocrinology (NIDE), Cairo, Egypt and was conforming to the ethical guidelines for research in humans. All subjects enrolled in this study provided written informed consent before their inclusion in the study. From January 2016 to December 2017, patients who had been referred to our outpatient clinic from community clinics for treatment of diabetes were consecutively recruited. The control (healthy) group included 8 subjects who were recruited from the general community during the same period of time.

All participants were recruited according to the following criteria: free-living, Caucasian, ages from 43–60 years, and normal values for blood count, hepatic function and renal creatinine tests. The T2DM patients of both diabetic groups were treated by oral hypoglycemic agents and/or insulin. None of them were complaining of any acute or chronic illness or neoplastic disease as confirmed by history taking from them. Exclusion criteria for both diabetic groups were hepatic diseases, renal diseases, or smokers.

The inclusion criteria for the control group were to be without any acute or chronic disease or clinical conditions involving the endocrine-metabolic system. Besides, they should neither be smokers nor alcoholics and not receiving any medications affecting the endocrine metabolic system (e.g. anti-thyroids, glucocorticosteroids).

#### Clinical evaluation

Personal history was taken including: onset and duration of diabetes, familial history of diabetes, history of diabetic coma and presence of any complication of diabetes. In addition, life style (cigarette smoking and physical activity) and present medication were checked and recorded. Anthropometric data collected as the weight and height were measured and then body mass index (BMI) was calculated as follows; BMI = Body weight in Kg/height in m^2^ **(*Bray GA*, *1987*)**[20] (S1 Table).

The inclusion criteria for T2DM+ATHR group (39 females) were to be diagnosed with T2DM at the beginning of the study and with (5–21 years) duration of the disease. Their age ranged from (43–57 years). BMI ranged from (24–39.1 kg/m^2^). The T2DM-ATHR (22 females) included, were diagnosed diabetics with (1–15 years) duration. Their age ranged from (43–60) years. BMI ranged from (22.2–37.1 kg/m^2^). The control group includes 8 females, apparently healthy subjects with matching socioeconomic status. Their age ranged from (43–51 years) and BMI ranged from (19.5–35.76 kg/m^2^).

### Material and methods

#### Blood samples collection

Venous blood sample (6 ml) was withdrawn from each subject after an overnight fasting (12 hours). The blood sample was divided into two tubes; 2 ml were added to EDTA coated tube to measure glycated hemoglobin (HbA1c) and 4 ml were allowed to clot at room temperature then centrifuged at 3000 rpm for 15 minutes and serum was separated.

#### Serum measurements

Sera were divided into two aliquots; one of them was stored at -20°C till assessment of SOST (using commercially available Human serum Sclerostin (SOST) ELISA kit Glory Science Co., Ltd, Catalog no.: 95081, USA), irisin (using commercially available Human serum Irisin ELISA kit Glory Science Co., Ltd, Catalog no.: 95512, USA) and serum Insulin concentration using commercially available Human serum Insulin ELISA kit DRG Diagnostics, Catalog no.: EIA-2935, DRG Diagnostics Instruments GmbH, Germany.

The other part was used immediately to assess fasting blood glucose and lipid profile (total cholesterol "T-C", HDL-C, LDL-C and triacylglycerol "TAG") using standard automated laboratory techniques. Both fasting glucose and insulin levels were used for assessment of insulin resistance by calculating Homeostasis Model Assessment (HOMA) index according to **(Levy *et al*., 1998)** [21]. HOMA-IR = fasting insulin (μIU/ml) X fasting glucose (mmol/L)/22.5.

Serum SOST was measured using a quantitative sandwich ELISA and the color change is measured spectrophotometrically at a wavelength of 450 nm. The concentration of SOST in the samples is then determined by comparing the optical density (O.D.) of the samples to the standard curve, they are reported throughout in picograms per mL (pg/mL) and the detection range is 80–4000 pg/mL.

Serum irisin was measured using a quantitative sandwich ELISA and the color change is measured spectrophotometrically at a wavelength of 450 nm. The concentration of irisin in the samples is then determined by comparing the O.D. of the samples to the standard curve, they are reported throughout in pg/mL and the detection range is 6–280 pg/mL.

#### Statistical analysis

Statistical analysis was performed using GraphInstat (version 3) and GraphPad (version 5) softwares. Continuous variables were presented as mean and standard error of mean (SE) for normally distributed variables while non-normally distributed variables were presented as mean and standard error of mean (SE) and their median and interquartile range (IQR) between brackets. The normality was checked for continuous variables using the Kolmogorov and Smirnov normality test. One-way ANOVA followed by Tukey-Kramer Multiple Comparisons Test were used to compare between more than 2 normally distributed groups while Kruskal-Wallis Test followed by Dunn’s Multiple Comparisons Tests for more than 2 groups that are non-normally distributed. Also, the Mann Whitney U test was used to compare between variables of 2 non-normally distributed groups. Pearson correlation coefficient (r) was calculated to explore the possible relationships between SOST and irisin and among them and other studied parameters. Statistical significance was acceptable at P value less than 0.05. The receiver operating characteristic (ROC) curve was used to examine the sensitivity and specificity of both SOST and irisin as potential markers for type 2 diabetes associated atherosclerosis where the area under the ROC curve (AUC) was measured. The ROC analysis shows the accuracy of the test which is measured by the AUC, S.E and P value. Post-hoc power analysis was calculated for both markers; SOST and irisin using clinicalc online available tool [22].

## Results

### Baseline characteristics of the study population

As expected by the inclusion criteria, there was a significant increase in FBG level of T2DM+ATHR group when compared to both T2DM-ATHR and control groups (P<0.001). Also, upon comparing the level of FBG of T2DM-ATHR with controls, there was a significant increase in the former group (P<0.01). Consistently, there was an extremely significant increase in HbA1c% on comparing both T2DM+ATHR and T2DM-ATHR diabetic groups to the control group (P<0.001 and P<0.01 respectively). The fasting insulin level in both T2DM+ATHR and T2DM-ATHR groups showed extremely significant increase in comparison to controls (P<0.001) (Table 1). Indeed, assessment of HOMA-IR revealed that T2DM+ATHR showed statistically significant increase in comparison to T2DM-ATHR (P<0.05) and controls (P<0.001) reflecting worsened fasting insulin resistance. Also, T2DM-ATHR showed significant increase in comparison to controls (P<0.01) (Table 1).

**Table 1:** Demographic, biochemical and clinical data for the studied groups.

The studied parameters	T2DM+ATHR	T2DM-ATHR	Control group	P- value
	Mean ± S.E	Mean ± S.E	Mean ± S.E	
**Age (Year)**^a^	49.95 ± 0.5(50–4)	47.59 ± 1(48–7)	45.63 ± 1.1(44.5–5)	0.0041
**Duration (Year)**^c^	12.05 ± 0.6(11–5)	8.25 ± 0.75(10–5)	—	0.0016
**BMI (Kg/m**^**2**^)^b^	31.4±0.53	29.1±0.82	26.99±1.6	0.0034
**Systolic BP (mmHg)**^a^	129.2±1.99 (130–20)	120±2.1 (120–20)	115±1.9 (115–10)	0.0016
**Diastolic BP (mmHg)**^a^	81±1.26 (80–10)	76.36±1.24 (80–10)	76.25±1.83 (80–10)	0.0389
**Total cholesterol (mg/dL)**^b^	243.5±8.2	220.09±8.03	206.25±12.94	0.0459
**Triacylglycerol(mg/dL)**^a^	199.7±19.17 (175–105)	139.7±11.72 (128–76)	92±12.28 (88.5–57.5)	0.001
**HDL-c (mg/dL)**^b^	42.9±1.2	43.5±1.8	47.6±2.3	0.3098
**LDL-c (mg/dL)**^b^	151.46±6.3	147.95±10.03	130.13±10.3	0.4131
**FBG (mg/dL)**^b^	281.07±14.27	197.5±13.7	97.25±2.37	< 0.0001
**HbA1c (%)**^a^	9.99±0.32 (9.4–2.2)	8.99±0.39 (8.8–2.1)	5.5±0.07 (5.6–0.2)	< 0.0001
**Fasting Insulin (uIU/mL)**^a^	14.38±1.07 (12.8–9.9)	12.65±0.96 (11.8–6.1)	4.29±1.05 (2.45–5.39)	< 0.0001
**HOMA**^a^	10.18±1.02 (8.79–5.6)	5.98±0.51 (6.1–3.16)	1.03±0.26 (0.55–1.2)	< 0.0001
**SOST (pg/mL)**^a^	259.9 ±17.98 (248.1–116.4)	165.8±13.12 (153.6–76.388)	142.0±13.31 (145.1–45.106)	P<0.0001
**Irisin (pg/mL)**^a^	32.91±2.545 (29.98–12.04)	58.55±13.19 (28.1–18.46)	473.6±112.7 (675.0–603.77)	P<0.0001

^a^Kruskall-Wallis test,

^b^One-way ANOVA test,

^C^ Mann Whitney U test

p<0.05 is significant. Normally and non-normally distributed results are provided in terms of mean ±S.E while for non-normally distributed results, their median and interquartile values are presented within brackets.

In addition, T2DM+ATHR cohort was significantly older compared to the control cohort (P<0.05) also there was a significant increase in the duration of the disease on comparing T2DM+ATHR cohort to T2DM-ATHR cohort (P<0.01). BMI of T2DM+ATHR were significantly higher compared to control group (P<0.01). The systolic blood pressure (systolic BP) of T2DM+ATHR cohort was significantly higher compared with T2DM-ATHR (P<0.05) and control (P<0.01) cohorts (Table 1).

The triacylglycerol levels of T2DM+ATHR cohort were significantly higher compared to control group (P<0.01) (Table 1). Although triacylglycerol of T2DM+ATHR group displayed higher levels compared to T2DM-ATHR group and T2DM-ATHR displayed higher levels compared to controls but these increments were not statistically significant (Table 1). There were no significant differences regarding total cholesterol, LDL-c and HDL-c among the studied groups (P>0.05).

### Serum SOST and irisin in T2DM-/+ATHR patients and controls

Serum SOST levels in T2DM+ATHR showed significant increase in comparison to T2DM-ATHR (power of 98.8%, P<0.001) and control (power of 100%, P<0.001) (Table 1). Also, elevated levels of serum SOST were also detected in T2DM-ATHR cohort compared to control cohort, yet such increment was not statistically significant (P>0.05). Conversely, serum irisin levels was remarkably significantly lower in both T2DM+ATHR cohort (power of 97.4%, P<0.001) and T2DM-ATHR cohort (power of 95.5%, P<0.01) in comparison to controls (Table 1). It is worth mentioning that further reduction in the mean of serum irisin level was observed in T2DM+ATHR cohort compared to T2DM-ATHR cohort though this decrease was not statistically significant (P>0.05) (Table 1).

**Fig 1:**
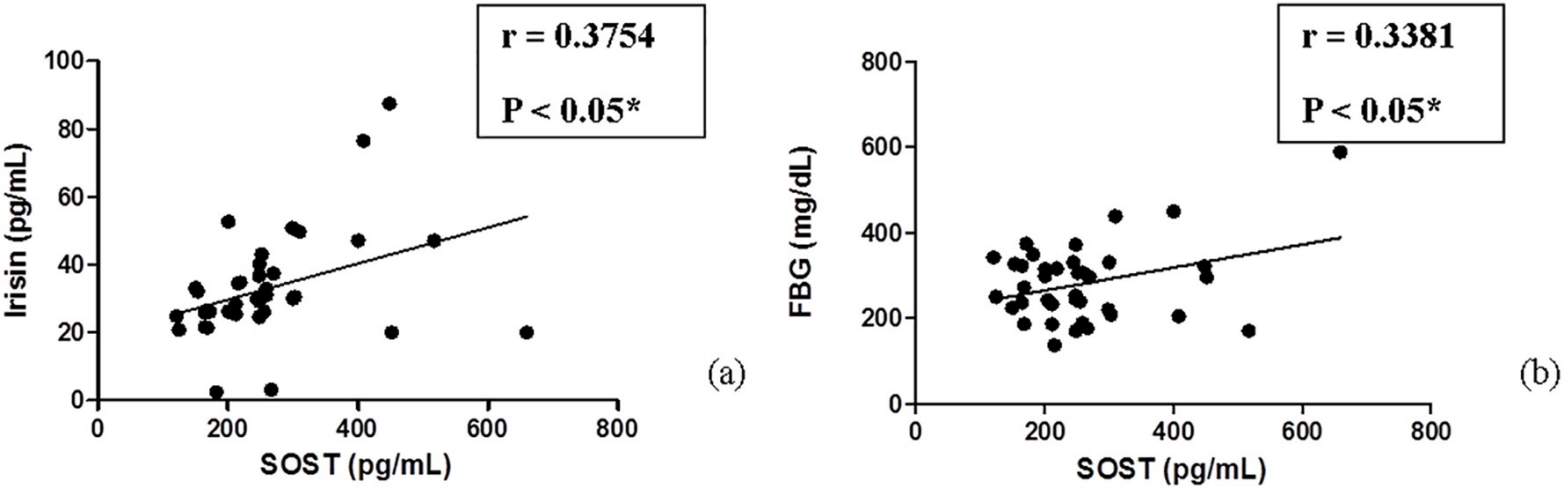
(a-b): Correlation between both SOST and each of irisin and FBG in the T2DM+ATHR diabetic group. (a) Significant positive correlation between SOST and irisin in type 2 diabetic group with atherosclerosis. (b) Significant positive correlation between SOST and FBG in type 2 diabetic group with atherosclerosis.

Importantly, the present study found a significant positive correlation between serum level of SOST and that of irisin in T2DM+ATHR group (95% Confidence Interval [0.06782–0.6178], P = 0.0185). Moreover, we noticed a significant positive correlation between SOST and FBG in T2DM+ATHR group (95% Confidence Interval [0.02516–0.5906], P = 0.0353). No other significant correlations were found (P>0.05) (Tables 2 and 3, Fig 1(a) and 1(b)).

**Table 2:** Correlation between both SOST and irisin and other studied parameters in T2DM+ATHR group.

The studied parameters	SOST (pg/mL)		Irisin (pg/mL)	
	Pearson r	P—value	Pearson r	P–value
**Age (Year)**	0.09866	0.5501	-0.05342	0.7467
**BMI (Kg/m**^**2**^)	-0.2590	0.1113	0.08063	0.6256
**Duration (years)**	-0.08497	0.6071	-0.0232	0.8884
**Systolic BP (mmHg)**	-0.05900	0.7213	0.03888	0.8142
**Diastolic BP (mmHg)**	0.01740	0.9163	0.1182	0.4737
**FBG (mg/dL)**	0.3381	0.0353	0.02927	0.8596
**HbA1c (%)**	-0.04418	0.7894	0.1828	0.2654
**Total cholesterol (mg/dL)**	-0.008548	0.9588	0.1469	0.3722
**Triacylglycerol (mg/dL)**	0.001758	0.9915	0.1611	0.3271
**HDL-C (mg/dL)**	-0.1591	0.3333	-0.0954	0.5634
**LDL-C (mg/dL)**	0.06856	0.6783	0.1078	0.5138
**Fasting Insulin (uIU/mL)**	-0.1377	0.4031	0.04097	0.8044
**HOMA**	-0.009117	0.9561	0.04009	0.8085
**Irisin (pg/mL)**	0.3754	0.0185	-----	-----
**SOST (pg/mL)**	-----	-----	0.3754	0.0185

**Table 3:** Correlation between both sclerostin and irisin and other studied parameters in T2DM-ATHR group.

The studied parameters	SOST (pg/mL)		Irisin (pg/mL)	
	Pearson r	P—value	Pearson r	P—value
**Age (Year)**	-0.002425	0.9915	-0.3026	0.1711
**BMI (Kg/m**^**2**^)	-0.09525	0.6733	-0.1354	0.5481
**Duration (years)**	0.07614	0.7363	-0.1526	0.4978
**Systolic BP (mmHg)**	0.1450	0.5197	-0.1083	0.6315
**Diastolic BP (mmHg)**	-0.03502	0.8771	0.4525	0.045
**FBG (mg/dL)**	-0.2350	0.2925	0.1767	0.4315
**HbA1c (%)**	0.1111	0.6225	-0.0001374	0.9995
**Total cholesterol (mg/dL)**	-0.04436	0.8446	0.09317	0.6801
**Triacylglycerol (mg/dL)**	-0.06387	0.7777	0.06716	0.7665
**HDL-C (mg/dL)**	0.2240	0.3163	0.05847	0.7960
**LDL-C (mg/dL)**	-0.2411	0.2797	-0.2639	0.2353
**Fasting Insulin (uIU/mL)**	-0.04783	0.8326	-0.05595	0.8047
**HOMA**	-0.1302	0.5635	0.1878	0.4025
**Irisin (pg/mL)**	0.3253	0.1396	-----	-----
**SOST (pg/mL)**	-----	-----	0.3253	0.1396

**Fig 2:**
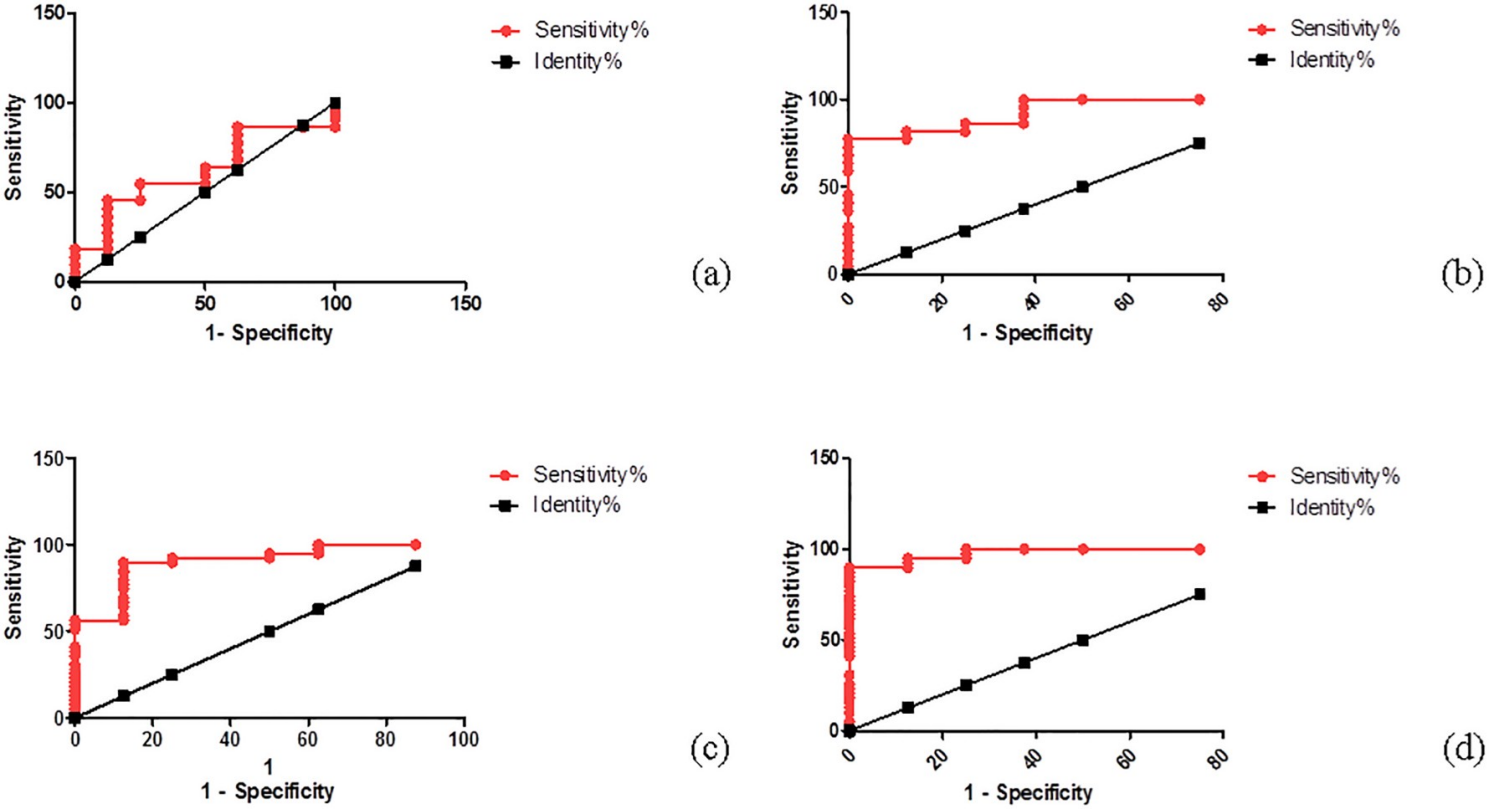
(a-d): ROC curve of both SOST and irisin in the 2 studied diabetic groups. (a) ROC curve of SOST in type 2 diabetic patients without atherosclerosis, (b) ROC curve of irisin in type 2 diabetic patients without atherosclerosis, (c) ROC curve of SOST in type 2 diabetic patients with atherosclerosis, and (d) ROC curve of irisin in type 2 diabetic patients with atherosclerosis.

ROC analysis of serum levels of irisin and SOST. It was found that AUC of ROC curve in T2DM-ATHR was 0.9318 for irisin with significant P-value 0.0003683(P < 0.001) while was 0.6193for SOST with P value 0.3247 (P > 0.05). Regarding the T2DM+ATHR group, it was found that AUC was 0.9808 for irisin with significant P-value P< 0.0001 while was 0.9071for SOST with significant P-value 0.0003275 (Table 4 and Fig 2(a)–2(d)).

**Table 4:** ROC curve analysis for SOST and irisin in T2DM+ATHR and T2DM-ATHR groups.

ROC curve analysis	T2DM+ATHR group			T2DM-ATHR group		
	AUC	S.E.	P—value	AUC	S.E.	P—value
**SOST (pg/mL)**	0.9071	0.0552	0.0003275	0.6193	0.1092	0.3247
**Irisin (pg/mL)**	0.9808	0.01724	< 0.0001	0.9318	0.0455	0.00037

## Discussion

In this study, all the recruited subjects were females, the mean value of the duration of diabetes and systolic blood pressure in the T2DM+ATHR were significantly higher than that of the T2DM-ATHR. Also, the mean value of age, BMI and systolic blood pressure of the T2DM+ATHR were significantly higher than the control cohort. These results are in accordance with **Rao et al., (2010)** who stated that long standing hyperglycemia causes many microvascular and macrovascular diseases including CVDs [2]. Additionally, **Rambhade *et al*., (2011)** stated that DM elevates the risk of both cardiac diseases and stroke and almost half DM patients are killed by CVDs. Also, there is a direct relationship between insulin resistance and being overweight that shall be translated into stemming of various diseases such as hypertension, T2DM and CVDs [23].

In the current study, the lipid profile showed significantly higher mean serum levels of triacylglycerol (TG) in T2DM+ATHR cohort compared to the control cohort. On the other hand, the total cholesterol and LDL-C of the T2DM+ATHR cohort were not significantly higher compared to the control group. With respect to the mean serum level of HDL-C, it was relatively lower in the T2DM+ATHR than in the T2DM-ATHR and the control groups. Moreover, the mean fasting insulin level was significantly higher in both diabetic groups compared to the control group. Consequently, the mean HOMA levels were significantly higher in both diabetic groups compared to the control group and in the T2DM+ATHR cohort compared to T2DM-ATHR cohort. FBG level and HbA1c% were significantly increased in both diabetic cohorts when compared to the controls. Also, FBG level was significantly increased in T2DM+ATHR cohort compared to T2DM-ATHR cohort. Consistent with the current lipid profile, it has been previously reported that dyslipidemia is higher in T2DM patients when compared to healthy subjects [24,25]. The underlying mechanism describing the pathogenesis of T2DM that promotes dyslipidemia occurrence is principally β-cell dysfunction and insulin resistance. It has been revealed that the increment in plasma TG levels is resulting from the weakened insulin ability to repress the production of large TG-rich VLDL (VLDL -TGs) in T2DM individuals. The weakened action of insulin on fat cells is deemed to be the cause of the defective suppression of the TGs breakdown inside the cells and the production of non-esterified (free) fatty acids (NEFAs) into the blood where the later elevated entry into the liver stimulates TG synthesis, thus, resulting in hypertriglyceridemia and post-prandial hyperlipidemia owing to the impaired activity of the lipoprotein lipase enzyme. Hypertriglyceridemia affects the blood coagulation via initiating thrombogenic changes. Besides, the increased VLDL-TGs levels reduce the cardio-protective HDL-C levels which leads to its lessened antioxidant and anti-atherogenic activities [26]. In addition, insulin resistance has been proven to play a crucial role in the emergence and progression of T2DM associated cardiovascular complications [27,28]. Also, **Haffner et al., (1997)** found that high insulin resistance assessed by HOMA-R characterized subjects with impaired glucose tolerance [29].

Given the fact that CVDs—driven by atherosclerosis- are one of the leading causes of mortality in patients with T2DM where women are at a substantially higher risk compared to men, identification of reliable sensitive predictive biomarkers is imperative. This study showed that the mean of serum SOST level in T2DM+ATHR cohort was significantly higher than T2DM-ATHR and control cohorts. Consistently, it has been lately suggested that SOST might act as an independent predictor of cardiovascular mortality in a population comprising subjects with and without T2DM[30]. Also, the mean of SOST of T2DM-ATHR cohort showed relative increase when compared to the control cohort. These results are in the same line with **García-Martín et al**., who reported increased circulating SOST level in T2DM independently of gender and age and its relevance with bone mass and turnover [31]. Notably, in a diabetic murine model scientists found high levels of SOST in calcified aorta tissues [32] and also in 3 human aortic samples from atherosclerotic patients[33]. Not only SOST is produced by osteocytes but also *in vitro* assays in a calcifying environment proved SOST production in VSMCs [34] that changed phenotypically to mineralizing osteoblast-like cells with the ability to express many bone forming genes among them the SOST gene. These findings elaborate a new implication of SOST on vascular pathology [3]. The suggested mechanism behind that is; since SOST is produced mainly by osteocytes and blocks the Wnt signaling pathway that is responsible for the subsequent blocking of adipogenesis via suppressing both the peroxisome proliferator-activated receptor-γ (PPARγ) and CCAAT/enhancer-binding protein-α (CEBPα) and blocking the thermogenic program through suppression of PPARγ coactivator-1α (PGC-1α); consequently, SOST stimulates adipogenesis which enhances the progression to T2DM and its associated complications [10]. This may imply its importance to be used as a future prognostic marker of the disease. Furthermore, it has been reported that serum SOST levels—which were significantly elevated in postmenopausal women—inversely associated with the circulating free estradiol index suggesting distinctive regulatory pattern of SOST production by estrogen within the female population [35]. Hence, it remains to be determined the effect of varying estradiol levels on SOST and their correlation with CVDs within pre/postmenopausal patients with T2DM.

In the current study, the means of irisin in both diabetic cohorts were significantly lower than that of the control cohort and the mean of irisin in T2DM+ATHR cohort was relatively lower than that of T2DM-ATHR cohort. In harmony with our findings, irisin levels were reported to be lower in patients with macrovascular disease (MVD) compared to those without MVD and the later was lower compared to healthy controls [16]. Thus, this study showed that irisin may act as a marker for macrovascular diseases in T2DM. In addition, r-Irisin increased the betatrophin expression which simultaneously stimulated the proliferation of pancreatic β-cell thus improves glucose tolerance [15]. **Xiang et al., (2014)** reported lower levels of circulating irisin in newly diagnosed T2DM without angiopathy compared to the controls in their study that investigated the relation between irisin levels and endothelial dysfunction [36]. Also, serum irisin was significantly reduced in both long-term and new-onset T2DM individuals compared with the non-diabetic controls [37,38]. This decrease in serum irisin can be attributed to its secretion in response to PGC-1α activation; this hormone induces browning of fat cells. This type of brown fat reduces body weight through increasing total body energy expenditure leading to an increase in the insulin sensitivity [10]. Additionally, in diabetic animal model, overexpression of irisin increased energy expenditure and decreased insulin resistance [17,39]. However, the effect of irisin in humans remains debated where **Norheim et al., (2014)** showed that irisin was slightly elevated in pre-diabetics compared to controls and long term training did not affect the plasma irisin levels [40]. So, collectively, the results of the studies on effect of irisin on the human subjects are contradictory [41].

The present study for the first time found a significant positive association between serum SOST and serum irisin levels in T2DM+ATHR cohort (P<0.05). Also, a significant positive association was found between serum SOST level and FBG in T2DM+ATHR cohort. However, no other associations were found with other studied parameters These results agreed with previously published studies which reported that there was no association between BMI and SOST [42,43]. On the other hand, it contradicted with the studies that showed positive correlation between BMI and SOST [44–46] and each of T2DM duration and HbA1c and SOST [31]. As regards to HOMA, the results agreed with **Yu et al., (2017)** who revealed that HOMA was weakly correlated with SOST levels [47]. Regarding irisin, the present results agreed with **Shoukry et al., (2016)** who concluded increased serum irisin levels in healthy subjects but decreased levels in T2DM patients [38]. Although this study found a positive correlation between SOST and irisin, this contradicts with the findings of **Colaianni et al., (2015)** which stated that administration of myokine irisin stimulated both the cortical bone mass and strength in mice and is followed by a significant increase in osteoblastic bone formation, up-regulated proosteoblastic genes, and reduced osteoblast inhibitors, such as SOST[48]. This may imply an undiscovered underlying cell-to-cell miscommunication that may occur in the course of the disease. We speculate that a feedback loop of regulatory cross-talks between SOST and irisin might account for this discrepancy. Nevertheless, further systematic studies are compulsory to reveal the functional and molecular underlying mechanisms.

This study has certain limitations. While the present study shows only correlation, the causal relationship between SOST and irisin remains to be determined. Secondly, the sample size is relatively small. Another potential limitation could be that we did not measure the physical activity indexes of the subjects enrolled. This may be a limitation since both SOST and irisin are influenced by physical activity. Strengths of our study comprises the evaluation of serum SOST and irisin levels in Egyptian T2DM+ATHR patients for the first time while focusing on the female population who are at greater risk and the examination of the presence of a correlation between the two markers and their sensitivity/specificity using ROC curve analysis as fundamental tools to evaluate their usefulness as novel reliable diagnostic and/or prognostic markers for the disease progression.

In conclusion, to the best of our knowledge, this study is the first to investigate the levels of SOST in Egyptian female patients with T2DM with and without atherosclerosis and the potential relation between SOST and irisin in the 2 studied T2DM cohorts. Indeed, there was a positive correlation between SOST and irisin in T2DM+ATHR cohort. Furthermore, SOST levels positively correlated with FBG in T2DM+ATHR cohort. Our data further support that Wnt signaling pathway is impaired in T2DM and that increased SOST and decreased irisin serum levels might cooperate together to trigger T2DM and worsen atherosclerosis as increasing SOST stimulates adipogenesis which aggravates T2DM and reducing irisin raises body weight. Finally, our data further support the value of using SOST and irisin as novel reliable diagnostic and/or prognostic biomarker of cardiovascular risk in patients with T2DM. Nonetheless, additional studies are required to investigate the potential cross-regulatory networks between SOST and irisin in T2DM and their relationship with glycemic control of this population.

## Supporting information

S1 Table Patients data of the studied cohorts. A table showing the anthropometric data, blood pressure measurements and medication history of the T2DM+ATHR, T2DM-ATHR and control cohorts.
(XLSX)

## Abbreviations

CVDs: cardiovascular diseases
DM: Diabetes mellitus
FBG: fasting blood glucose
HOMA: Homeostasis Model Assessment
MSC: mesenchymal stem cells
PPARγ: peroxisome proliferator-activated receptor-γ
ROC: receiver operator characteristic
SOST: sclerostin
T2DM: type II diabetes mellitus
T2DM+ATHR: type 2 diabetes patients with atherosclerosis
T2DM-ATHR: type 2 diabetes patients without atherosclerosis
TAG: triacylglycerol
T-C: total cholesterol
VSMC: vascular smooth muscle cells

## References

[1] Zheng Y, Ley SH, Hu FB. Global aetiology and epidemiology of type 2 diabetes mellitus and its complications. Nat Rev Endocrinol 2017 10.1038/nrendo.2017.151 29219149

[2] Rao MU, Sreenivasulu M, Chengaiah B, Reddy KJ, C C. Herbal Medicines for Diabetes Mellitus: A Review. Int J Pharm Tech Res. 2010;2: 1883–1892.

[3] Morales-Santana S, García-Fontana B, García-Martín A, Rozas-Moreno P, García-Salcedo JA, Reyes-García R, et al Atherosclerotic disease in type 2 diabetes is associated with an increase in sclerostin levels. Diabetes Care. 2013;36: 1667–1674. 10.2337/dc12-1691 23288857 PMC3661830

[4] Norhammar A, Schenck-Gustafsson K. Type 2 diabetes and cardiovascular disease in women. Diabetologia. 2013 10.1007/s00125-012-2694-y 22945305

[5] Madonna R and De Caterina R. Cellular and molecular mechanisms of vascular injury in diabetes disease in diabetes. Vasc Pharmacol. 2011;54: 68–74. 10.1016/j.vph.2011.03.005 21453786

[6] Wang X, Xiao Y, Mou Y, Zhao Y, Blankesteijn WM H J. A role for the β-catenin/T-cell factor signaling cascade in vascular remodeling. Circ Res. 2002;90: 340–347. 11861424 10.1161/hh0302.104466

[7] Couffinhal T D P and D C. Beta-catenin nuclear activation: common pathway between Wnt and growth factor signaling in vascular smooth muscle cell proliferation? Circ Res. 2006;99: 1287–1289. 10.1161/01.RES.0000253139.82251.31 17158343

[8] Tsaousi A, Williams H, Lyon CA, Taylor V, Swain A, Johnson JL G S. Wnt4/β-catenin signaling induces VSMC proliferation and is associated with intimal thickening. Circ Res. 2011;108: 427–436. 10.1161/CIRCRESAHA.110.233999 21193738

[9] Catalano A, Pintaudi B, Morabito N, Di Vieste G, Giunta L, Lucia Bruno M, et al Gender differences in sclerostin and clinical characteristics in type 1 diabetes mellitus. Eur J Endocrinol. 2014;171: 293–300. 10.1530/EJE-14-0106 24891138

[10] Klangjareonchai T, Nimitphong H, Saetung S, Bhirommuang N, Samittarucksa R, Chanprasertyothin S, et al Circulating Sclerostin and Irisin Are Related and Interact with Gender to Influence Adiposity in Adults with Prediabetes. Int J Endocrinol. Hindawi Publishing Corporation; 2014;2014: 1–6. 10.1155/2014/261545 25276128 PMC4167818

[11] Poole KE, van Bezooijen RL, Loveridge N, Hamersma H, Papapoulos SE, Löwik CW, R J. Sclerostin is a delayed secreted product of osteocytes that inhibits bone formation. FASEB J. 2005;19: 1842–1844. 10.1096/fj.05-4221fje 16123173

[12] van Bezooijen RL, ten Dijke P, Papapoulos SE, L C. SOST/sclerostin, an osteocyte-derived negative regulator of bone formation. Cytokine Growth Factor Rev. 2005;16: 319–327. 10.1016/j.cytogfr.2005.02.005 15869900

[13] Christodoulides C, Lagathu C, Sethi JK and V-P A. “Adipogenesis and WNT signalling,.” Trends Endocrinol Metab. 2009;20: 16–24. 10.1016/j.tem.2008.09.002 19008118 PMC4304002

[14] Urano T, Shiraki M, Ouchi Y I S. Association of circulating sclerostin levels with fat mass and metabolic disease—related markers in Japanese postmenopausal women. J Clin Endocrinol Metab. 2012;97: E1473–E1477. 10.1210/jc.2012-1218 22639287

[15] Colaianni G, Mongelli T, Colucci S, Cinti S, Grano M, Colaianni G, Mongelli T, Colucci S, Cinti S G M. Crosstalk Between Muscle and Bone Via the Muscle-Myokine Irisin. Curr Osteoporos Rep. Current Osteoporosis Reports; 2016;14: 132–137. 10.1007/s11914-016-0313-4 27299471

[16] Zhang M, Chen P, Chen S, Sun Q, Zeng QC, Chen JY, Liu YX, Cao XH, Ren M W J. The association of new inflammatory markers with type 2 diabetes mellitus and macrovascular complications: a preliminary study. Eur Rev Med Pharmacol Sci. 2014;18: 1567–1572. 24943964

[17] Boström P, Wu J, Jedrychowski MP, Korde A, Ye L, Lo JC, Rasbach KA, Boström EA, Choi JH, Long JZ, Kajimura S, Zingaretti MC, Vind BF, Tu H, Cinti S, Højlund K, Gygi SP S B. A PGC1-α-dependent myokine that drives brown-fat-like development of white fat and thermogenesis. Nature. 2012;481: 463–468. 10.1038/nature10777 22237023 PMC3522098

[18] BM S. Banting Lecture 2012: Regulation of adipogenesis: toward new therapeutics for metabolic disease. Diabetes,. 2013;62: 1774–1782. 10.2337/db12-1665 23704518 PMC3661621

[19] Zhang Y, Mu Q, Zhou Z, Song H, Zhang Y, Wu F, et al Protective effect of irisin on atherosclerosis via suppressing oxidized low density lipoprotein induced vascular inflammation and endothelial dysfunction. PLoS One. 2016;11: e0158038 10.1371/journal.pone.0158038 27355581 PMC4927070

[20] Bray GA and Gray DS. Obesity. Part I- pathogenesis. West J Med. 1988;149: 429–441. 3067447 PMC1026489

[21] Levy JC M D and H M. Correct homeostasis model assessment (HOMA) evaluation uses the computer program. Diabetes Care. 1998;21: 2191–2192. 9839117 10.2337/diacare.21.12.2191

[22] Kane. SP. Post. ClinCalc [Internet]. [cited 20 Aug 2018]. http://clincalc.com/stats/Power.aspx.

[23] Rambhade S, Singh S, Goswami R R A. Occurrence, Complications, and Interventions of Diabetes: A New Understanding of an Old Problem. Syst Rev Pharm. 2011;2: 8–18.

[24] Ismail IS, Nazaimoon W, Mohamad W, Letchuman R, Singaraveloo M, Hew FL, Shuguna C K B. Ethnicity and glycaemic control are major determinants of diabetic dyslipidaemia in Malaysia. Diabet Med. 2001;18: 501–508. 11472471 10.1046/j.1464-5491.2001.00494.x

[25] Jacobs MJ, Kleisli T, Pio JR, Malik S, L’Italien GJ, Chen RS W N. Prevalence and control of dyslipidemia among persons with diabetes in the United States. Diabetes Res Clin Pract. 2005;70: 263–269. 10.1016/j.diabres.2005.03.032 15890427

[26] Vijayaraghavan K. Treatment of dyslipidemia in patients with type 2 diabetes. Lipids Health Dis. 2010;9: 144–156. 10.1186/1476-511X-9-144 21172030 PMC3022752

[27] Bonora E, Tessari R, Micciola R, Zenere M, Targher G, Padovani R, Falezza G M M. Intimal medial thickness of the carotid artery in non diabetic and NIDDM subjects: relationship with insulin resistance. Diabetes Care. 1997;20: 627–631. 9096992 10.2337/diacare.20.4.627

[28] Yip J F F and R G. Resistance to insulin mediated glucose disposal as a predictor of cardiovascular disease. J Clin Endocrinol Metab. 1998;83: 2773–2776. 10.1210/jcem.83.8.5005 9709945

[29] Haffner SM M H and S M. The homeostasis model in the San Antonio Heart Study. Diabetes Care. 1997;20: 1087–1092. 9203442 10.2337/diacare.20.7.1087

[30] Novo-Rodríguez C, García-Fontana B, Luna-Del Castillo JD, Andújar-Vera F, Ávila-Rubio V, García-Fontana C, Morales-Santana S, Rozas-Moreno P, M-T M. Circulating levels of sclerostin are associated with cardiovascular mortality. PLoS One. 2018;13: 1–14. 10.1371/journal.pone.0199504 29928063 PMC6013204

[31] García-Martín A, Rozas-Moreno P, Reyes-García R, Morales-Santana S, García-Fontana B, García-Salcedo JA, et al Circulating levels of sclerostin are increased in patients with type 2 diabetes mellitus. J Clin Endocrinol Metab. 2012;97: 234–241. 10.1210/jc.2011-2186 22031520

[32] Shao JS, Cheng SL, Pingsterhaus JM, Charlton-Kachigian N, Loewy AP, T DA. Msx2 promotes cardiovascular calcification by activating paracrine Wnt signals. J Clin Invest. 2005;115: 1210–1220. 10.1172/JCI24140 15841209 PMC1077175

[33] Didangelos A, Yin X, Mandal K, Baumert M, Jahangiri M M M. Proteomics characterization of extracellular space components in the human aorta. Mol Cell Proteomics. 2010;9: 2048–2062. 10.1074/mcp.M110.001693 20551380 PMC2938114

[34] Zhu D, Mackenzie NC, Millán JL, Farquharson C M V. The appearance and modulation of osteocyte marker expression during calcification of vascular smooth muscle cells. PLoS One. 2011;6: e19595 10.1371/journal.pone.0019595 21611184 PMC3096630

[35] Mirza FS, Padhi ID, Raisz LG, Lorenzo JA. Serum sclerostin levels negatively correlate with parathyroid hormone levels and free estrogen index in postmenopausal women. J Clin Endocrinol Metab. 2010; 10.1210/jc.2009-2283 20156921 PMC2853994

[36] Xiang L, Xiang G, Yue L, Zhang J, Z L. Circulating irisin levels are positively associated with endothelium-dependent vasodilation in newly diagnosed type 2 diabetic patients without clinical angiopathy. Atherosclerosis. 2014;235: 328–333. 10.1016/j.atherosclerosis.2014.04.036 24911636

[37] Liu JJ, Wong MD, Toy WC, Tan CS, Liu S, Ng XW, Tavintharan S, Sum CF L S. Lower circulating irisin is associated with type 2 diabetes mellitus. J Diabetes Complications. 2013;27: 365–369. 10.1016/j.jdiacomp.2013.03.002 23619195

[38] Shoukry A, Shalaby SM, El-Arabi Bdeer S, Mahmoud AA, Mousa MM, Khalifa A. Circulating serum irisin levels in obesity and type 2 diabetes mellitus. IUBMB Life. 2016; 544–556. 10.1002/iub.1511 27220658

[39] Zhou X, Li R, Liu X, Wang L, Hui P, Chan L, et al ROCK1 reduces mitochondrial content and irisin production in muscle suppressing adipocyte browning and impairing insulin sensitivity. Sci Rep. Nature Publishing Group; 2016;6: 29669 10.1038/srep29669 27411515 PMC4944137

[40] Norheim F, Langleite TM, Hjorth M, Holen T, Kielland A, Stadheim HK, Gulseth HL, Birkeland KI, Jensen J D C. The effects of acute and chronic exercise on PGC-1 alpha, irisin and browning of subcutaneous adipose tissue in humans. FEBS J. 2014;281: 739–749. 10.1111/febs.12619 24237962

[41] Panati K, Suneetha Y, Narala VR. Irisin/FNDC5—An updated review. Eur Rev Med Pharmacol Sci. 2016;20: 689–697. 26957272

[42] Arasu A, Cawthon PM, Lui LY, Do TP, Arora PS, Cauley JA, Ensrud KE C S. Serum sclerostin and risk of hip fracture in older Caucasian women. J Clin Endocrinol Metab. 2012;97: 2027–2032. 10.1210/jc.2011-3419 22466341 PMC3387417

[43] Thorson S, Prasad T, Sheu Y, Danielson ME, Arasu A, Cummings SR C J. Sclerostin and bone strength in women in their 10th decade of life. J Bone Miner Res. 2013;28: 2008–2016. 10.1002/jbmr.1929 23505206 PMC3723747

[44] Amrein K, Amrein S, Drexler C, Dimai HP, Dobnig H, Pfeifer K, Tomaschitz A, Pieber TR, F-P A. Sclerostin and its association with physical activity, age, gender, body composition, and bone mineral content in healthy adults. J Clin Endocrinol Metab. 2012;97: 148–154. 10.1210/jc.2011-2152 21994959

[45] Sheng Z, Tong D, Ou Y, Zhang H, Zhang Z, Li S, et al Serum sclerostin levels were positively correlated with fat mass and bone mineral density in Central South Chinese postmenopausal women. Clin Endocrinol (Oxf). 2012;76: 797–801. 10.1111/j.1365-2265.2011.04315.x 22151063

[46] Szulc P, Boutroy S, Vilayphiou N, Schoppet M, Rauner M, Chapurlat R, Hamann C H L. Correlates of bone microarchitectural parameters and serum sclerostin levels in men: the STRAMBO study. J Bone Miner Res. 2013;28: 1760–1770. 10.1002/jbmr.1888 23408601

[47] Yu OHY, Richards B, Berger C, Josse RG, Leslie WD, Goltzman D, et al The association between sclerostin and incident type 2 diabetes risk: a cohort study. Clin Endocrinol (Oxf). 2017;86: 520–525. 10.1111/cen.13300 28090669

[48] Colaianni G, Cuscito C, Mongelli T, Pignataro P, Buccoliero C, Liu P, et al The myokine irisin increases cortical bone mass. Proc Natl Acad Sci. 2015;112: 12157–12162. 10.1073/pnas.1516622112 26374841 PMC4593131

